# 

**Published:** 1900-02

**Authors:** 


***NOTICE.—This JOURNAL and THE INTERNATIONAL JOURNAL OF SURGERY one year for $1.00 cash, to new subscribers. No out-of-town checks accepted unless 10c. is added for cost of cashing.***

